# Willingness and Motivation of Saudi Patients with Keratoconus to Participate in Clinical Trials

**DOI:** 10.7759/cureus.17580

**Published:** 2021-08-30

**Authors:** Shahd A Al Mahfud, Ali A Al Saeed, Rakan J Alsahly, Faris H Binyousef, Norah S Alshabib, Nada A Alyousef, Ahmed A AlRubaian

**Affiliations:** 1 Medicine, Imam Mohammad Ibn Saud Islamic University, Riyadh, SAU; 2 Medicine, King Faisal University, Al Hofuf, SAU; 3 College of Medicine, Majmaah University, Al Majma'ah, SAU; 4 Medicine, King Saud University, Riyadh, SAU

**Keywords:** keratoconus, clinical research, participants, willingness, barriers

## Abstract

Introduction

Keratoconus is a bilateral non-inflammatory ectasia, characterized by well-described histopathological changes such as stromal thinning, epithelial iron deposition, and breaks in Bowman’s layer. The success of clinical intervention among patients with keratoconus is widely determined by randomized clinical trials, and despite associated difficulties, such trials may improve vision and quality of life.

Aim

This study aimed to assess the willingness of patients with keratoconus in Saudi Arabia to undergo clinical trials. We further aimed to identify patients’ beliefs and attitudes towards clinical trials and to establish possible barriers to trial recruitment, potentially improving the quality of future clinical trials and research.

Materials and methods

This was a quantitative analytical cross-sectional study conducted between October 2020 and March 2021 among patients with keratoconus in Saudi Arabia. A self-administered questionnaire was distributed among the targeted patients. The questionnaire identified the socio-demographic characteristics of the patients and included questions on willingness, motivation, potential barriers, and helpful resources. All statistical analyses were performed using SPSS version 21 (IBM Corp, Armonk, USA).

Results

A total of 462 patients were recruited. The most common age group was 16-25 years (39.8%). There were slightly more females (51.3%) than males (48.7%). The prevalence of patients with a previous history of keratoconus was 36.8%. Results revealed that 37.2% of the patients had great motivation to take part in clinical research, and 22.3% indicated a high score in potential barriers to participation, whereas nearly 48% showed a high score in helpful resources. Statistical tests revealed that being in an older age group, having children, and possessing a higher monthly income were factors associated with increased barriers to participation in clinical research.

Conclusion

Patients with keratoconus showed great motivation to participate in clinical research studies and provided helpful resources. The knowledge that participation could benefit others was a primary motivator, while encouragement from other patients who participated in clinical research was the main helpful resource. Possible side effects were shown to be the major concern of the patients.

## Introduction

Keratoconus is a bilateral non-inflammatory ectasia characterized by well-described histopathological changes such as stromal thinning, epithelial iron deposition, and breaks in Bowman’s layer. It leads to corneal protrusion, which in turn causes myopic astigmatism and, ultimately, an impairment in visual quality [1]. A previous report considered the willingness of patients presenting with advanced glaucoma to participate in a clinical trial [2], and a further study concluded that 73% of patients with chronic eye disease were prepared to enter a clinical trial [3]. However, no previous study has assessed the willingness of patients with keratoconus to enter clinical trials, and previous epidemiological studies on keratoconus in Saudi Arabia are limited. For example, a study conducted in Asir Province, Saudi Arabia concluded that the incidence of keratoconus in that province was high, as reflected by an incidence of 20 cases per 100,000 people [4]. The success of clinical interventions among patients with keratoconus is widely determined by randomized clinical trials, and despite associated difficulties, such trials may improve vision and quality of life [5]. These difficulties have been highlighted by varying recruitment problems; for example, only (31%) of trials have reached their recruitment planned target, and an extension was granted for half (53%). Early recruitment problems were also reported in 77 (63%) trials [6]. Understanding patient factors that encourage, or hinder patients may influence the success of patient recruitment into clinical trials. A lack of motivation and negative opinions about clinical research were among the most significant reasons why patients may not be willing to participate. However, other factors were significant in the decision to participate, such as the likelihood of improving one's own vision and helping others improve theirs [2].

Therefore, by assessing the willingness of patients with keratoconus in Saudi Arabia to undergo clinical trials, we aim to identify patients’ beliefs and attitudes towards clinical trials and establish possible barriers to trial recruitment, while potentially improving the quality of clinical trials and research.

## Materials and methods

Methods

This cross-sectional study was conducted to estimate the prevalence of patients with keratoconus in Saudi Arabia willing to participate in a clinical trial. It was shown by a previous study that 73% of patients with chronic eye disease were willing to participate in a clinical trial [3]. Using the single proportion sample size calculation formula, a confidence level of 95% and a precision of 5%, a sample size of 303 participants was needed. Taking into consideration the 30% non-response rate, our sample size was estimated to be around 394. The study was conducted on patients with keratoconus within Saudi Arabia’s hospitals during the period from October 2020 to March 2021. We included all keratoconus patients who were pre-diagnosed with keratoconus by ophthalmologists in Saudi Arabia. Minor patients defined by age less than 16 years were excluded. 

Statistical analysis

Statistical Package for Social Sciences, version 21 (IBM Corp, Armonk, USA) was the statistical software used to analyze the data in this project. The factors that influence participation in clinical research studies among keratoconus patients were evaluated using three dimensions: motivating factors (10 items), potential barriers (seven items), and helpful resources (seven items), where 5-point Likert scale categories ranging from “No motivation/barrier/helpful” coded as 0 to “most motivation/barrier/helpful” coded as 4 were the answer options. The total score for each dimension was achieved by adding all the items related to motivating factors, potential barriers, and helpful resources. The higher the score, the higher the influence of each dimension on the patient, and by using 50% and 75% of the total score as cutoff points, the level of motivation factors, potential barriers, and helpful resources was determined. Patients were classified as low level if the score was 50% or below, 50% to 75% of the total score was viewed as average level, and above 75% was considered high level.

Descriptive statistics were presented as numbers, percentages (%), mean, and standard deviation whenever appropriate. Between comparisons, a Chi-square test, Mann-Whitney U test, or Kruskal-Wallis test was applied. Statistical collinearity was measured by using a Kolmogorov-Smirnov test, plus a Shapiro-Wilk test. The overall motivation, barriers, and helpful resources scores followed an abnormal distribution; thus, non-parametric tests were applied. A p-value of <0.05 was considered statistically significant.

Ethical considerations 

Institutional Review Board approval was obtained from Imam Mohammad Ibn Saud Islamic University Institutional Review Board (IRB number 63 - 2021). The data were collected by a self-administered questionnaire that was given to patients with keratoconus. Informed consent form was obtained from each participant, and those who did not sign the informed consent documentation were excluded.

## Results

For this study, 462 patients were recruited. Table 1 presents the socio-demographic characteristics of the patients, with or without a previous history of keratoconus. The most common age group was 16-25 years (39.8%), where more than half were female (51.3%), and approximately 57% were unmarried. With respect to educational level, 57.8% had a bachelor’s degree, and 44.6% were employed. Moreover, 43.7% had less than 5,000 SAR (Saudi Riyal) of monthly income, and 67.4% of patients had children. It was revealed that age group in years (p<0.001), gender (p=0.002), marital status (p=0.043), having children (p=0.014), occupational status (p<0.001), and monthly income (p=0.001) showed significant relationships with a history of keratoconus.

**Table 1:** Socio-demographic characteristics of the patients according to a history of keratoconus § P-value has been calculated using a Chi-square test. ** Significant at p<0.05 level.

Study variables	Overall N (%) ^(n=462)^	History of keratoconus		P-value ^§^
		Yes N (%) ^(n=170)^	No N (%) ^(n=292)^	
Age group				
16–25 years	184 (39.8%)	29 (17.1%)	155 (53.1%)	<0.001 **
26–35 years	153 (33.1%)	100 (58.8%)	53 (18.2%)	
36–45 years	76 (16.5%)	35 (20.6%)	41 (14.0%)	
>45 years	49 (10.6%)	06 (03.5%)	43 (14.7%)	
Gender				
Male	225 (48.7%)	99 (58.2%)	126 (43.2%)	0.002 **
Female	237 (51.3%)	71 (41.8%)	166 (56.8%)	
Marital status				
Married	200 (43.3%)	84 (49.4%)	116 (39.7%)	0.043 **
Unmarried	262 (56.7%)	86 (50.6%)	176 (60.3%)	
Having children				
Yes	173 (37.4%)	76 (44.7%)	97 (33.2%)	0.014 **
No	289 (62.6%)	94 (55.3%)	195 (66.8%)	
Educational level				
High school or below	109 (23.6%)	32 (18.8%)	77 (26.4%)	0.191
Diploma holder	46 (10.0%)	21 (12.4%)	25 (08.6%)	
Bachelor’s degree	267 (57.8%)	100 (58.8%)	167 (57.2%)	
Master’s or PhD	40 (08.7%)	17 (10.0%)	23 (07.9%)	
Occupational status				
Employed	206 (44.6%)	91 (53.5%)	115 (39.4%)	<0.001 **
Unemployed	131 (28.4%)	66 (38.8%)	65 (22.3%)	
Student	125 (27.1%)	13 (07.6%)	112 (38.4%)	
Monthly income (SAR)				
None	67 (14.5%)	37 (21.8%)	30 (10.3%)	0.001 **
<5,000	202 (43.7%)	57 (33.5%)	145 (49.7%)	
5,000–10,000	103 (22.3%)	39 (22.9%)	64 (21.9%)	
>10,000	90 (19.5%)	37 (21.8%)	53 (18.2%)	

Table 2 shows the overall health of patients and their willingness to participate in clinical research. Following the results, it was observed that approximately two-thirds (33.8%) declared an excellent perceived overall health rating. The prevalence of patients who had previously participated in clinical research was 6.3%. Of those, 4.5% had participated in at least one clinical research study and 1.7% indicated more than one. Furthermore, 32.5% would prefer to participate if they were paid, while 31.4% indicated that payment did not matter. When comparing to the history of keratoconus, it was found that overall health perceived showed a positive relationship with the history of keratoconus (p<0.001).

**Table 2:** Patients’ overall health and willingness to participate in clinical research according to a history of keratoconus § P-value has been calculated using a Chi-square test. ** Significant at p<0.05 level.

Study variables	Overall N (%) ^(n=462)^	History of keratoconus		P-value ^§^
		Yes N (%) ^(n=170)^	No N (%) ^(n=292)^	
Overall health perceived rating				
Excellent	156 (33.8%)	37 (21.8%)	119 (40.8%)	<0.001 **
Very good	145 (31.4%)	46 (27.1%)	99 (33.9%)	
Good	111 (24.0%)	51 (30.0%)	60 (20.5%)	
Fair	28 (06.1%)	16 (09.4%)	12 (04.1%)	
Poor	22 (04.8%)	20 (11.8%)	02 (0.70%)	
Previous participation in a clinical research study				
Yes	29 (06.3%)	11 (06.5%)	18 (06.2%)	0.896
No	433 (93.7%)	159 (93.5%)	274 (93.8%)	
Frequency of previous participation in clinical research for the last 5 years				
None	433 (93.7%)	159 (93.5%)	274 (93.8%)	0.991
One	21 (04.5%)	08 (04.7%)	13 (04.5%)	
More than one	08 (01.7%)	03 (01.8%)	05 (01.7%)	
Would you prefer to participate in clinical research if you were paid?				
Yes	150 (32.5%)	52 (30.6%)	98 (33.6%)	0.535
No	167 (36.1%)	67 (39.4%)	100 (34.2%)	
Does not matter	145 (31.4%)	51 (30.0%)	94 (32.2%)	

Table 3 describes the factors that influenced the patients to participate in clinical research. The factors were divided into three dimensions: motivating factors, potential barriers, and helpful resources. For motivating factors, the top three statements where patients demonstrated high ratings were “Knowledge gained from my participation will benefit someone in the future” (mean: 3.49), followed by “How well the research study is explained to me” (mean: 3.37) and “Doctor’s reputation in the community” (mean: 3.27). For potential barriers, patients showed high ratings in the following three statements: “Risk of unknown side effects” (mean: 3.05), followed by “Multiple follow-up visits related to the study” (mean: 2.47), and “My distrust in doctors” (mean: 2.46). Finally, for helpful resources, high ratings were noted in the following statements: “Having opportunity to speak to a patient who has participated in a clinical research” (mean: 2.93), “Having all material provided in my own language” (mean: 2.91), and “Having access to a medical interpreter throughout the study” (mean: 2.90).

**Table 3:** Factors that influence participation in clinical research studies a Response has a range from 0 = No motivation to 4 = Most motivating factor. b Response has a range from 0 = No barrier to 4 = Greatest barrier c Response has a range from 0 = No help to 4 = Most help resources.

Motivating Factors ^a^	Mean ± SD
Knowledge gained from my participation will benefit someone in the future	3.49 ± 0.92
How well the research study is explained to me	3.37 ± 1.03
Doctor’s reputation in the community	3.27 ± 1.13
Good relationship with my doctor	3.23 ± 1.24
The doctor conducting the research speaks the same language as me	2.57 ± 1.58
A friend or family member participating in the same study	2.51 ± 1.52
Money offered for my participation	2.08 ± 1.60
My desire to please the doctor	2.06 ± 1.55
The doctor conducting the research is the same race/ethnicity/religion/language as me	1.92 ± 1.64
The doctor conducting the research is the same gender (sex) as me	1.80 ± 1.65
Total Score	26.3 ± 9.29
Potential Barriers ^b^	
Risk of unknown side effects	3.05 ± 1.30
Multiple follow-up visits related to the study	2.47 ± 1.34
My distrust in doctors	2.46 ± 1.51
Time commitment	2.30 ± 1.39
Clinical research studies are too hard to understand	2.11 ± 1.39
My family’s concern	1.97 ± 1.53
My religious beliefs	1.87 ± 1.66
Total Score	16.2 ± 6.28
Helpful resources ^c^	
Having opportunity to speak to a patient who has participated in clinical research	2.93 ± 1.24
Having all material provided in my own language	2.91 ± 1.29
Having access to a medical interpreter throughout the study	2.90 ± 1.28
Having access to a support group of patients who have participated in clinical research	2.89 ± 1.24
Written material explaining the research study	2.82 ± 1.29
I would like to participate if there is a friend or family member who is participating in the same study	2.66 ± 1.37
DVDs or electronic material explaining the research study	2.52 ± 1.38
Total Score	19.6 ± 7.23

**Figure 1:**
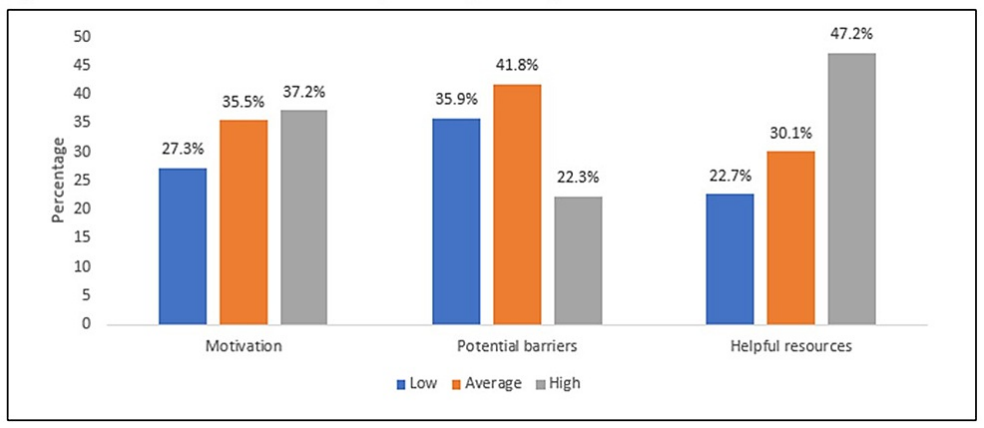
Level of factors in motivation, barriers, and helpful resources

Figure 1 depicts the level of motivation, potential barriers, and helpful resources. It can be observed that the level of motivation was low, average, and high (27.3%, 35.5%, and 37.2%, respectively), while in potential barriers, low, average, and high levels were detected as 35.9%, 41.8%, and 22.3%, respectively. In helpful resources, 47.2%, 30.1%, and 22.7% were classified as high, average, and low levels.

A Pearson correlation coefficient has been performed in Table 4 to determine the linear relationship between motivating factors, potential barriers, and helpful resources. Based on the results, it was found that the correlation between motivating factors and potential barriers was positively highly statistically significant (r=0.185; p<0.001). It was also observed that the correlation between motivating factors and helpful resources was also positively highly statistically significant (r=0.524; p<0.001). Finally, a positively highly statistically significant correlation was observed between potential barriers and helpful resources (r=0.154; p<0.001).

**Table 4:** Correlation (Pearson-R) between scores of motivation, barriers and helpful resources ** Correlation is significant at the 0.01 level (2-tailed).

Variable	Motivating factor	Potential barriers	Helpful resources
Motivating factors	1		
Potential barriers	0.185 **	1	
Helpful resources	0.524 **	0.154 **	1

When measuring the difference in the scores of motivating factors, potential barriers, and helpful resources with regard to the socio-demographic characteristics of the patients, it was found that the median scores of potential barriers were statistically significantly higher in the older age group (>30 years) (Z=-2.106; p=0.035), those with children (Z=-2.366; p=0.018) and those who had monthly earnings of 5,000 SAR or more (X2=8.114; p=0.017). On the other hand, the median scores of helpful resources were statistically significantly higher for those who had a diploma or below (Z=-2.214; p=0.027) and those with a previous history of keratoconus (Z=-2.051; p=0.040). The difference in the median scores of motivating factors was not statistically significant when compared to the socio-demographic characteristics of the patients (p>0.05) (Table 5).

**Table 5:** Statistical difference between the scores of motivation, potential barriers and helpful resources in relation to the socio-demographic characteristics of the patients a P-value has been calculated using Mann Whitney U test. b P-value has been calculated using Kruskal Wallis test. ** Significant at p<0.05 level. IQR: inter-quartile range.

Factor	Motivating Score (40) Median (IQR)	Barriers Score (28) Median (IQR)	Helpful Score (28) Median (IQR)
Age group ^a^			
≤30 years	25.7 (14.00)	16.0 (09.25)	21.0 (10.00)
>30 years	26.0 (12.00)	17.0 (09.75)	21.0 (12.00)
Z-test; P-value	-1.058; 0.290	-2.106; 0.035 **	-1.159; 0.247
Gender ^a^			
Male	26.0 (12.50)	17.0 (09.00)	21.0 (10.00)
Female	27.0 (13.00)	17.0 (09.00)	22.0 (11.00)
Z-test; P-value	-0.638; 0.524	-1.480; 0.139	-1.470; 0.142
Marital status ^a^			
Married	26.0 (12.00)	17.0 (08.75)	21.0 (11.00)
Unmarried	27.0 (15.00)	16.0 (10.25)	21.0 (11.00)
Z-test; P-value	-1.402; 0.161	-1.187; 0.235	-0.574; 0.566
Having children ^a^			
Yes	27.0 (13.00)	17.0 (07.50)	21.0 (11.00)
No	26.0 (14.00)	16.0 (11.00)	21.0 (11.00)
Z-test; P-value	-0.836; 0.403	-2.366; 0.018 **	-0.084; 0.933
Educational level ^a^			
Diploma or below	27.0 (12.00)	16.0 (09.00)	22.0 (10.00)
Bachelor’s degree or higher	26.0 (13.00)	17.0 (09.00)	21.0 (11.00)
Z-test; P-value	-1.305; 0.192	-1.269; 0.205	-2.214; 0.027 **
Occupational status ^b^			
Employed	26.0 (13.00)	17.0 (10.00)	21.0 (09.00)
Unemployed	28.0 (14.00)	16.0 (08.00)	22.0 (12.00)
Student	26.0 (12.00)	17.0 (09.50)	21.0 (11.00)
X2-test; P-value	1.145; 0.564	0.506; 0.776	4.071; 0.131
Monthly income (SAR) ^b^			
None	27.0 (15.00)	16.0 (12.00)	22.0 (10.00)
<5,000	26.0 (13.00)	16.0 (09.00)	21.0 (11.00)
≥5,000	27.0 (13.00)	17.0 (09.50)	21.0 (12.00)
X2-test; P-value	0.424; 0.809	8.114; 0.017 **	2.997; 0.223
Overall health perceived rating ^b^			
Excellent	27.0 (16.00)	16.5 (09.00)	22.0 (10.75)
Very good	26.0 (11.50)	16.0 (08.00)	20.0 (09.50)
Good	26.0 (12.00)	18.0 (10.00)	21.0 (09.00)
Fair or poor	27.0 (11.50)	16.5 (10.25)	22.5 (12.50)
X2-test; P-value	5.057; 0.168	1.440; 0.696	4.143; 0.246
Previous participation in a clinical research ^a^			
Yes	23.0 (13.00)	16.0 (14.50)	22.0 (09.00)
No	27.0 (13.00)	17.0 (09.00)	21.0 (11.00)
Z-test; P-value	-0.167; 0.868	-0.029; 0.977	-1.134; 0.257
Previous history of keratoconus ^a^			
Yes	26.0 (13.00)	17.0 (10.00)	22.0 (09.00)
No	27.0 (14.00)	17.0 (09.00)	20.0 (11.00)
Z-test; P-value	-1.800; 0.072	-0.288; 0.773	-2.051; 0.040 **

## Discussion

Clinical trials are the foundation of medical development, and they are the most essential method of finding an advanced medical treatment. Literature suggests that failure to achieve the required sample size in a clinical trial would cast doubt on the validity of the clinical research and even more in a randomized controlled trial [7-8]. The purpose of the present study is to evaluate the willingness and motivation of Saudi patients with keratoconus to participate in clinical research studies.

In this study, the most common motivating factor of the patients was a “selfless act (altruism)” or participation in clinical research that would benefit others in the future. This result is consistent with the study of Jones et al. [8]. They reported that altruism was the greatest motivation of the patients, which was similarly reported by Detoc and colleagues [9], as well as Gaul and associates [10]. Another published study conducted in the U.K. [2] indicated that patients’ willingness to participate in randomized clinical studies was directly attributed to their level of comprehension and insight about the medical condition, its treatment, and the research process. This is comparable with our results showing the great motivation of keratoconus patients was dependent on how well the research is explained. In Saudi Arabia [11], research indicated that the majority of patients were motivated to participate in clinical trials for religious reasons and because they were approached by their primary physicians. In China [12], the most common reason for willingness to participate in research was the respondents’ desire for new treatment and their trust in hospitals and doctors. In our study, patients were also motivated to participate in clinical trials due to a good relationship with their doctor and his or her reputation in the community, which was in line with previous findings. Furthermore, we then calculated the overall motivation score of the patients based on 10 items for motivational factors. According to our results, the overall mean motivation score was 26.3 (SD 9.29) out of 40 points, and based on the given criteria, 37.2% had high scores, 35.5% had average scores, and 27.3% were classified with low scores. This indicates that the overall motivation of patients to participate in a clinical trial was positive. Similarly, Altaf and colleagues [13], documented that 69% of the patients who were scheduled for surgery were positive towards clinical trial participation. Alataf et al. [13] also pointed out that educational levels were observed to have a significant association with willingness to participate in clinical trials (p<0.05), while Kong and associates [12] noted that patients who have a spouse or children were more likely to participate in clinical trials. However, in our study, we observed that the motivation scores in all socio-demographic characteristics were not significantly different across the groups (p>0.05), which was inconsistent with previous reports.

Helpful resources are another factor that might influence patients to participate in clinical research. In our study, the willingness of patients to be involved in clinical trials will improve if they can talk to other patients who have participated in previous research and therefore learn more about the research processes. Willingness to participate may also increase if the materials provided were in the patients’ own language. Other important helpful resources to increase the chance of participation were the availability of a medical interpreter throughout the course of the study; access to a group of patients who had previously participated in a clinical trial; correct documentation of the clinical research, both hard and electronic copies; and having a close family member in the same study. These views are in accordance with the study of Leighton et al. [2]. Based on their reports, the recruitment rate may be enhanced by ensuring that patients have full and accurate information about treatment alternatives and that uncertainty exists for best patient outcomes between treatment options and reassuring potential participants that the research process will not compromise medical care. Furthermore, we then measured the overall helpful resources score of the patients. Based on the given criteria, the overall mean helpful resources score was 19.6 (SD 7.23) out of 28 points with 47.2% considered as high score, 30.1% considered as average and 22.7 % considered as low score. Our findings also revealed that patients who were diploma holders or below and those with a previous history of keratoconus were associated with an increased score in helpful resources. That is, we can surmise that less education and a diagnosis of keratoconus would be influential factors in the willingness to participate in clinical trial studies.

Our study verified previous findings that potential side effects could play a big factor in participating in clinical research [9-12]. Further barriers to participation were multiple follow-up visits, mistrust of the doctor, time commitment, and a lack of knowledge about the study. On the other hand, the patients did not see family commitment and religious beliefs as barriers to participating in the clinical trials. In Canada [14], reports indicated that general apprehension of entering clinical trials and the fear of experimentation were the major barriers to participation in research, which was not consistent with our reports. Likewise, we measured the overall scores of potential barriers based on the given statements (seven items). Based on the results, the overall mean barrier score was 16.2 (SD 6.28) out of 28 points with approximately 42% classified as average score, 35.9% were low and 22.3% were considered as high score. Similarly, we observed that an older age group (>30 years), having children, and being a high earner were the factors associated with an increased score to barrier. Albeit these groups of patients were likely to exhibit hesitation to participate in clinical trials.

Moreover, it can be observed that the linear relationship between motivating factors, potential barriers, and helpful resources was positive and highly statistically significant (p<0.001). This indicates that the increase in the score of motivation will also increase the scores of potential barriers and helpful resources and vice versa, which could be the supplemental findings of this study.

It is important to note that the prevalence of patients who were previously diagnosed with keratoconus was 6.3% with a positive relationship with age group, gender, marital status, having children, occupational status, monthly income, and overall health. In addition, 6.3% reported previous participation in clinical trials. Literature suggests that patients were not accustomed to clinical research participation as previously reported in Saudi Arabia [11,13], Canada [8], and Germany [10]. However, when asked if they preferred to receive incentives to join the research, 32.5% of them responded affirmatively, while 31.4% had no firm opinion, and the rest opposed it (36.1%).

## Conclusions

Great motivation and helpful resources have been observed among patients with keratoconus with a view to participating in clinical research studies. Knowing that participation could benefit others was a great motivator while encouragement from other patients who participated in clinical research was the main helpful resource. However, potential side effects were the major concern reported by the patients. Being older, having a child, and earning more resulted in greater concerns regarding participation, while being less educated and having a previous history of keratoconus were the greater helpful resources in motivating them to join clinical research. Furthermore, awareness of clinical trials participation is necessary. Motivational-based factors and helpful resources should be utilized more to encourage more patients to partake in clinical trials. Thus, decreasing the impact of suppressing factors will enhance the chance of clinical trial participation among patients with keratoconus.

## References

[1] Romero-Jiménez M, Santodomingo-Rubido J, Wolffsohn JS (2010). Keratoconus: a review. Cont Lens Anterior Eye.

[2] Leighton P, Lonsdale AJ, Tildsley J, King AJ (2012). The willingness of patients presenting with advanced glaucoma to participate in a trial comparing primary medical vs primary surgical treatment. Eye (Lond).

[3] Wilmarth J, Gardner T (1956). Willingness of patients with chronic eye disease to participate in clinical trials. Invest Ophthalmol Vis Sci.

[4] Assiri AA, Yousuf BI, Quantock AJ, Murphy PJ (2005). Incidence and severity of keratoconus in Asir province, Saudi Arabia. Br J Ophthalmol.

[5] Chew EY (2011). The value of randomized clinical trials in ophthalmology. Am J Ophthalmol.

[6] McDonald AM, Knight RC, Campbell MK (2006). What influences recruitment to randomised controlled trials? A review of trials funded by two UK funding agencies. Trials.

[7] Friedman DB, Bergeron CD, Foster C, Tanner A, Kim SH (2013). What do people really know and think about clinical trials? A comparison of rural and urban communities in the South. J Community Health.

[8] Jones JM, Nyhof-Young J, Moric J, Friedman A, Wells W, Catton P (2006). Identifying motivations and barriers to patient participation in clinical trials. J Cancer Educ.

[9] Detoc M, Launay O, Dualé C (2019). Barriers and motivations for participation in preventive vaccine clinical trials: experience of 5 clinical research sites. Vaccine.

[10] Gaul C, Malcherczyk A, Schmidt T, Helm J, Haerting J (2010). [Motivation of patients to participate in clinical trials. An explorative survey]. Med Klin (Munich).

[11] Al-Dakhil LO, Alanazy R, AlHamad RE, Al-Mandeel H, Alobaid A (2016). Attitudes of patients in developing countries toward participating in clinical trials: a survey of Saudi patients attending primary health care services. Oman Med J.

[12] Kong Q, Mei H, Lai Y, Shi S, Li Y, He L, Qin HY (2017). Barriers and facilitators to participation in clinical trial among lymphoma patients from Sun Yat-sen University Cancer Center in China: an observation study. Medicine (Baltimore).

[13] Altaf A, Bokhari R, Enani G ( 2019). Patients' attitudes and knowledge toward clinical trial participation. Saudi Surg J.

[14] Cheung AM (2008). Barriers and motivations for women to participate in cardiovascular trials. J Obstet Gynaecol Can.

